# Successful delivery in a cornual pregnancy after expectant management with traditional Chinese medicine and low-molecular-weight heparin: A case report

**DOI:** 10.1097/MD.0000000000040446

**Published:** 2024-11-08

**Authors:** Xiao Lin, Yizhou Fu, Richu Lin, Xiaqin Cai, Mingli Zhang

**Affiliations:** a Department of Ultrasound, Wenzhou Medical University, Wenzhou, Zhejiang Province, China; b Department of Ultrasound, Kunming Medical University, Hangzhou, Zhejiang Province, China; c Department of Dermatology, Tongde Hospital of Zhejiang Province, Hangzhou, Zhejiang Province, China; d Department of Ultrasound, Tongde Hospital of Zhejiang Province, Hangzhou, Zhejiang Province, China.

**Keywords:** cornual pregnancy, ectopic pregnancy, expectant management, fertility, interstitial pregnancy

## Abstract

**Rationale:**

Horn pregnancy is a rare subtype of ectopic pregnancy that presents a diagnosis and treatment challenge due to its nonspecific symptoms and high risk of rupture.

**Patient concerns:**

A 32-year-old woman without vaginal pregnancy with history of right corner who presented with painless vaginal bleeding.

**Diagnoses:**

A transvaginal ultrasound revealed a pregnancy sac implanted in the left corner of the uterus, confirming the diagnosis of a cornual pregnancy.

**Interventions:**

Treatment options include pharmacological or surgical interventions, and anticipatory treatment is rarely recommended. Conservative treatment was chosen after extensive consultation, and the patient was treated with low molecular weight heparin and traditional Chinese medicine.

**Outcomes:**

Subsequent ultrasound tests showed stable fetal development and a successful cesarean section.

**Lessons:**

This case highlights the success of combining low molecular weight heparin with traditional Chinese medicine in the treatment of cornual pregnancy. Factors affecting horn pregnancy, diagnostic challenges, and treatment considerations are discussed. Further research is necessary to determine the best management strategy and to ensure safe delivery for patients with impaired fertility but a strong desire to conceive.

## 1. Introduction

Cornual pregnancy is a rare subtype of ectopic pregnancy in which the gestational sac implants in the interstitial segment of the fallopian tube. It accounts for 2% to 4% of ectopic pregnancies and carries high risks of rupture and hemorrhage, as well as a mortality rate ranging from 2.0% to 2.5%.^[1]^ Abnormal transport of the fertilized ovum is widely accepted as the possible mechanism, with other risk factors including in vitro fertilization (IVF), uterine anomalies, previous ectopic pregnancies or pelvic surgeries, intrauterine device use, and chronic or acute inflammatory processes.^[2,3]^

Early diagnosis of cornual pregnancy can be challenging because of its nonspecific clinical symptoms, making imaging studies the most reliable modalities for differentiation. Ultrasound is the most commonly used modality, whereas magnetic resonance imaging can be an option, although less frequently employed.^[2,4]^ Common ultrasound findings include an eccentric location of the gestational sac within the uterus, with minimal or absent myometrium around the lateral aspect of the sac.^[5]^ The “interstitial line sign,” characterized by an echogenic line between the gestational sac and endometrial cavity, can also aid in diagnosis.^[2]^ Three-dimensional sonography has also demonstrated clinical value.^[6]^

Treatment options should be individualized according to specific patient goals and needs. Pregnancy termination is usually recommended because of the high risk of rupture, which can be managed either medically with methotrexate or surgically if indicated.^[7,8]^ Although expectant management is not frequently advised, there have been reports of successful delivery.^[9–11]^ In this study, we report a case of successful delivery in a cornual pregnancy after conservative management with traditional Chinese medicine (TCM) and low-molecular-weight heparin (LMWH).

## 2. Case presentation

A 32-year-old Chinese nulligravida presented to the clinic with a 1-day history of painless vaginal bleeding. Her last menstrual period was 45 days before presentation. She had a history of right laparoscopic salpingotomy because of cornual pregnancy in 2020. Otherwise, she denied any pertinent medical, surgical, social, or family history. On pelvic exam, a small amount of blood-tinged secretion was present in the vaginal canal, and the uterus was anteverted with cervical cylindrical and mild columnar epithelial displacement. There were no tender masses on palpation.

**Figure 1.**
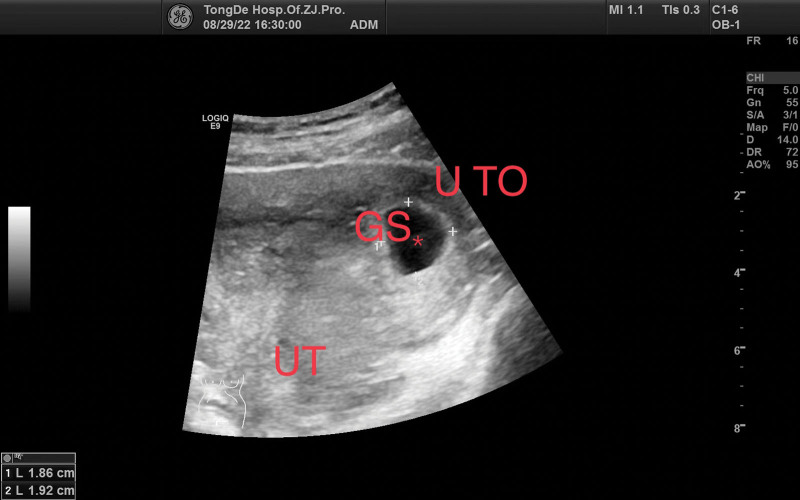
Gestational ultrasound before treatment (6 weeks, 3 days).

**Figure 2.**
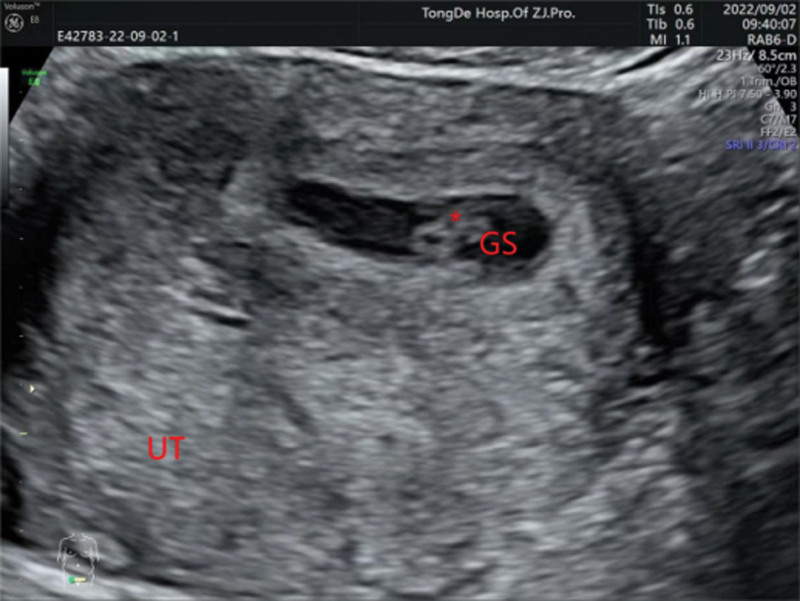
Gestational ultrasound after 3 days of combined treatment (6 weeks, 6 days).

**Figure 3.**
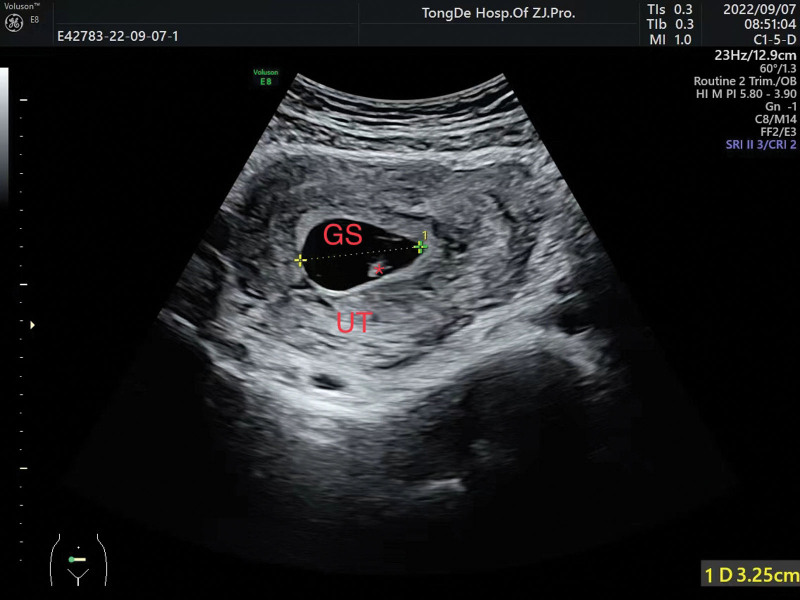
Gestational ultrasound after 8 days of combined treatment (7 weeks, 4 days).

The initial laboratory examination revealed a human chorionic gonadotropin level of 1135.15 mIU/mL. Transvaginal ultrasound revealed a 2.2 × 1.9 × 1.5-cm^3^ gestational sac in the left uterine cornua with an identifiable yolk sac and a 0.26-cm embryo, and primitive cardiac activity was also observed (Fig. 1). A diagnosis of cornual pregnancy was made. The patient and her partner wanted to avoid future IVF and preferred to not sacrifice the remaining functional fallopian tube. After extensively counseling them on the risks of rupture and hemorrhage, they chose expectant management and provided written informed consent. Because there were no standard treatments regimen recommended, the patient received subcutaneous LMWH 4100 IU twice daily and a combined TCM regimen consisting of dodder seed, Loranthi Ramulus et Folium, stir-fried Atractylodes rhizome, Szechuan lovage rhizome, Baikal skullcap root, red sage root, stir-fried Aurantii Fructus, dried tangerine peel, Perilla stem, pyritum, notoginseng root, Pulsatillae Radix, Chinese angelica root, and licorice root. After 3 days of treatment, the vaginal bleeding had improved significantly, and a repeat ultrasound was performed. A 0.65-cm embryo remained in the left uterine cornua with prominent primitive cardiac activity (Fig. 2). At week 7 of gestation, ultrasound revealed that the size of gestational sac had increased to 3.59 × 3.32 × 1.49 cm^3^, and the embryo had grown to 0.77 cm with prominent primitive cardiac activity, indicating appropriate development and growth of the fetus (Fig. 3).

All medications were discontinued after 8 days of treatment, the patient no longer had any vaginal bleeding or abdominal pain, and she was advised to receive regular follow-up at a high-risk obstetric clinic. During the return obstetric visits, the patient and the baby experienced no complications throughout the remainder of the pregnancy. She successfully delivered a healthy female fetus via cesarean section (CS) at 40 weeks, 3 days.

The study received approval from the Ethics Committee of Tongde Hospital of Zhejiang Province (No. 067). Patient informed consent has been obtained.

## 3. Discussion

Cornual pregnancy, also known as interstitial pregnancy, represents a rare and challenging variant of ectopic pregnancy characterized by the implantation of a gestational sac within the interstitial segment of the fallopian tube. The diagnosis and management of cornual pregnancy pose significant clinical challenges because of its nonspecific presentation and high risks of rupture and hemorrhage. In our case, the diagnosis was made promptly, and extensive communication about the goal of care with the patient and her family ensured successful delivery after expectant management with LMWH and a combined TCM regimen.

There are several risk factors that might contribute to cornual pregnancy, including a previous history of ectopic pregnancy, ovulation induction, previous ipsilateral or bilateral salpingectomy, IVF-assisted conception, and a history of sexually transmitted infections.^[12]^ Bayyarapu and Gundabattula^[13]^ conducted a retrospective cohort study of 35 patients with interstitial pregnancy. Their results revealed that prior ipsilateral salpingectomy is a unique risk factor for interstitial pregnancies, and it was observed in one-fifth of their cohort.^[13]^ Hiersch et al^[14]^ investigated 24 patients diagnosed with cornual pregnancy, and 11 of them conceived via assisted reproductive techniques. In our case, the patient underwent a right salpingectomy because of previous right cornual pregnancy, increasing her risks of another cornual pregnancy. However, the risk factors for recurrent interstitial pregnancy remain unclear, and future studies are needed to address this issue to permit better communication between physicians and their patients.

The diagnosis of cornual pregnancy can often be delayed or missed because of its nonspecific clinical symptoms, including vaginal bleeding and abdominal pain. As highlighted in our case and other studies, transvaginal ultrasound remains the cornerstone for diagnosing cornual pregnancy, enabling visualization of the gestational sac and identifying the location.^[15–18]^ Despite advances in imaging detection, only approximately 40% of the cases reported in the literature were diagnosed before rupture, and some patients still needed laparotomy or laparoscopic surgeries to make the final diagnosis.^[12]^ Our patient had evident sonographic findings to reach a diagnosis before progressing to rupture, making expectant management a possible option.

The management of cornual pregnancy necessitates a tailored approach based on individual patient goals and clinical considerations. Conventional treatment options typically include medical or surgical intervention to mitigate the risks of rupture and hemorrhage. Although expectant management is not a common recommendation, it has resulted in successful deliveries in some reported cases. Sentilhes et al^[9]^ reported a case of successful CS delivery in a cornual pregnancy after expectant management in a patient who received multiple cycles of IVF. Fernandez et al^[19]^ treated their patients with either an injection of potassium chloride or expectant management, and they reported 3 safe deliveries after conservative management in their cohort. Our patient and her family strongly desired conservative management, and given her stable hemodynamics, we agreed to proceed medically and forgo emergent surgical options. As the use of TCM during pregnancy is extremely common in Chinese patients, we incorporated a TCM regimen, which aimed to improve fetal blood supply for better growth and development.

In conclusion, our case demonstrates that expectant management with TCM combination therapy might be a safe and successful option in a selected group of patients with cornual pregnancy. Future studies should investigate strategies to ensure safe deliveries in patients with compromised fertility but a strong desire for conception.

## Author contributions

**Conceptualization:** Xiao Lin, Yizhou Fu.

**Data curation:** Xiao Lin, Yizhou Fu.

**Formal analysis:** Xiao Lin, Yizhou Fu.

**Methodology:** Richu Lin, Xiaqin Cai.

**Project administration:** Richu Lin, Xiaqin Cai.

**Resources:** Richu Lin, Xiaqin Cai.

**Software:** Richu Lin, Xiaqin Cai.

**Supervision:** Richu Lin, Xiaqin Cai.

**Validation:** Richu Lin, Xiaqin Cai.

**Visualization:** Richu Lin, Xiaqin Cai.

**Writing – original draft:** Xiao Lin.

**Writing – review & editing:** Xiao Lin, Xiaqin Cai, Mingli Zhang.
